# Evaluation of the relationship between phantom position and computed tomography dose index in cone beam computed tomography when assuming breast irradiation

**DOI:** 10.1002/acm2.13282

**Published:** 2021-05-28

**Authors:** Hiroyuki Ueno, Kosuke Matsubara, Akihiro Takemura, Masato Hizume, Sayuri Bou

**Affiliations:** ^1^ Department of Radiology Takaoka City Hospital Takaoka Toyama Japan; ^2^ Division of Health Sciences, Graduate School of Medical Sciences Kanazawa University Kanazawa Ishikawa Japan; ^3^ Department of Radiotherapy Takaoka City Hospital Takaoka Toyama Japan

**Keywords:** computed tomography dose index, cone beam computed tomography, image‐guided radiation therapy

## Abstract

This study aims to investigate the influence of the phantom position on weighted computed tomography dose index (CTDI_w_) in cone beam computed tomography (CBCT) when assuming breast irradiation. Computed tomography dose index (CTDI) was measured by the x‐ray volume imaging of CBCT using parameters for image‐guided radiation therapy (IGRT) in right breast irradiation. The measurement points of CTDI ranged from 0 (center) to 16 cm in the right–left (RL) direction, and from 0 (center) to 7.5 cm in the anterior–posterior (AP) direction, which assumed right breast irradiation. A nonuniform change exists in the relative value of CTDI_w_ when the phantom deviated from the isocenter of CBCT. The CTDI_w_ was ~30% lower compared with the value at the isocenter of CBCT when the phantom deviated 7.5 and 16 cm at the AP and RL directions, respectively. This study confirmed the influence of the phantom position on the CTDI values of CBCT. The CTDI measured at the isocenter of CBCT overestimates that measured at the irradiation center of the breast.

## 1 INTRODUCTION

In recent years, image‐guided radiation therapy (IGRT) with cone beam computed tomography (CBCT) using an electronic portal imaging device has been extensively used in radiotherapy. Many studies have reported that IGRT with CBCT is useful for improving setup errors and observing changes in tumor volume.^1^, ^2^, ^3^, ^4^ IGRT can compensate for position misalignment by capturing images before irradiation. However, because CBCT uses radiation, its radiation exposure may cause cancer and other health problems. Several reports exist that correlate CBCT doses with cancer risks.^5^, ^6^, ^7^, ^8^, ^9^, ^10^, ^11^ Therefore, knowing and maintaining the IGRT dose as low as possible is essential. One of the dose indexes for CBCT is CT dose imdex (CTDI). Generally, the center of the torso is adjusted to the gantry isocenter in diagnostic CT, and the CTDI is measured by placing a phantom at the gantry isocenter. In CBCT for IGRT, the center of the patient torso may not be at the isocenter of CBCT, for example, the isocenter of CBCT is located within the planning target volume and the center of the trunk is not adjusted to the isocenter of CBCT in the breast irradiation. Therefore, the center of the torso of the patient does not coincide with the center of CBCT. No detailed report exists on the influence of phantom location when measuring CTDI although certain studies^12^, ^13^ are available on dosimetry of CBCT for IGRT.

ICRP Publication 103 dictates that the effective dose can be easily estimated by multiplying the dose length product (DLP) using the conversion factor.^14^ DLP is calculated from weighted CTDI (CTDI_w_) and longitudinal direction irradiation range. In breast irradiation, the center of CBCT is at the center of the radiotherapy site, and the center of the torso is not at the center of CBCT. Estimating the effective dose from DLP derived from CTDI_w_ measured at the isocenter of CBCT may not be suitable in this situation because it is not the radiation dose received at the actual location. This study examined the influence of phantom positioning on CTDI values when assuming breast irradiation.

## 2 MATERIALS AND METHODS

### 2.A X‐ray volume imaging CBCT system

The CTDI of CBCT using x‐ray volume imaging (XVI) was measured. The XVI is a device attached to the Elekta Synergy (Elekta, Stockholm, Sweden). The system comprises an x‐ray tube and a flat panel detector. These components are perpendicular to the gantry of the linear accelerator. Moreover, the XVI is available for CBCT, fluoroscopy, and radiography.

### 2.B Phantom and dosimeter

**Fig. 1:**
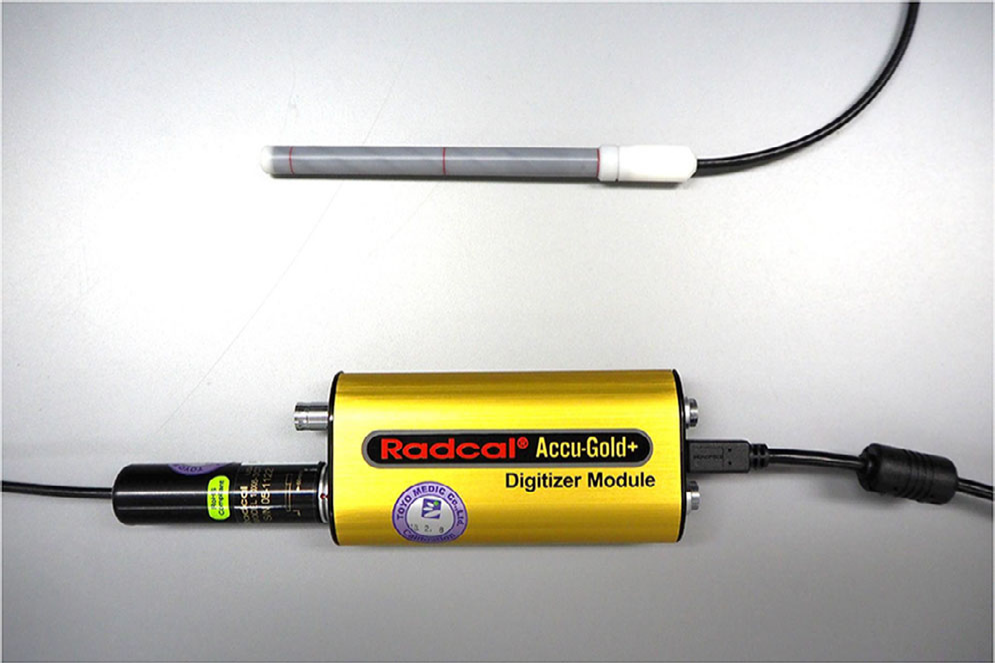
A 10‐cm ionization chamber and a digitizer module for CTDI measurement.

A cylindrical acrylic phantom was used for the CTDI measurement. This phantom had a diameter of 32 cm, a longitudinal length of 15 cm, a hole in the center, and four holes (top, bottom, left, and right) in the periphery that were located 1 cm inside the surface of the phantom. The dosimeters used were ACCU‐GOLD+ (Radcal, Monrovia, CA, USA) and an ionization chamber (10X6‐3CT: Radcal) that has an active length 10 cm. Figure 1 shows the dosimeters used in this study.

### 2.C Acquisition parameters

This study measured the CTDI of CBCT at the location that assumed right breast irradiation. One of the methods for reducing breast dose from CBCT in IGRT is to irradiate the x rays from the back. In the system used in this study, the collimator of the gantry head, which is placed vertically to CBCT imaging system, protrudes toward the isocenter. Thus, the collimator may interfere with the patient which limits the methods of dose reduction compared with the left breast irradiation when CBCT is performed on the right breast by irradiating x rays from the back of the patient. In the facility of the first author, CBCT is not performed by irradiating x rays from the back of the patient for right breast irradiation in consideration of patient safety. Therefore, the dose of CBCT in right breast irradiation is expected to be higher compared with the left breast irradiation. Thus, CBCT dose was investigated when assuming right breast irradiation.

The acquisition parameters were a tube voltage of 100 kV, a tube current of 20 mA per frame, 5.5 frames per second collection, and irradiation angle of XVI from 265° to 110° with clock‐wise direction. The collimator was S20, which yielded an axial field‐of‐view of 27 cm, and no additional filter was attached (F0). The maximum diameter of the reconstruction was 270 mm, and the longitudinal x‐ray beam width was 276.7 mm.

### 2.D Measurement geometry

**Fig. 2:**
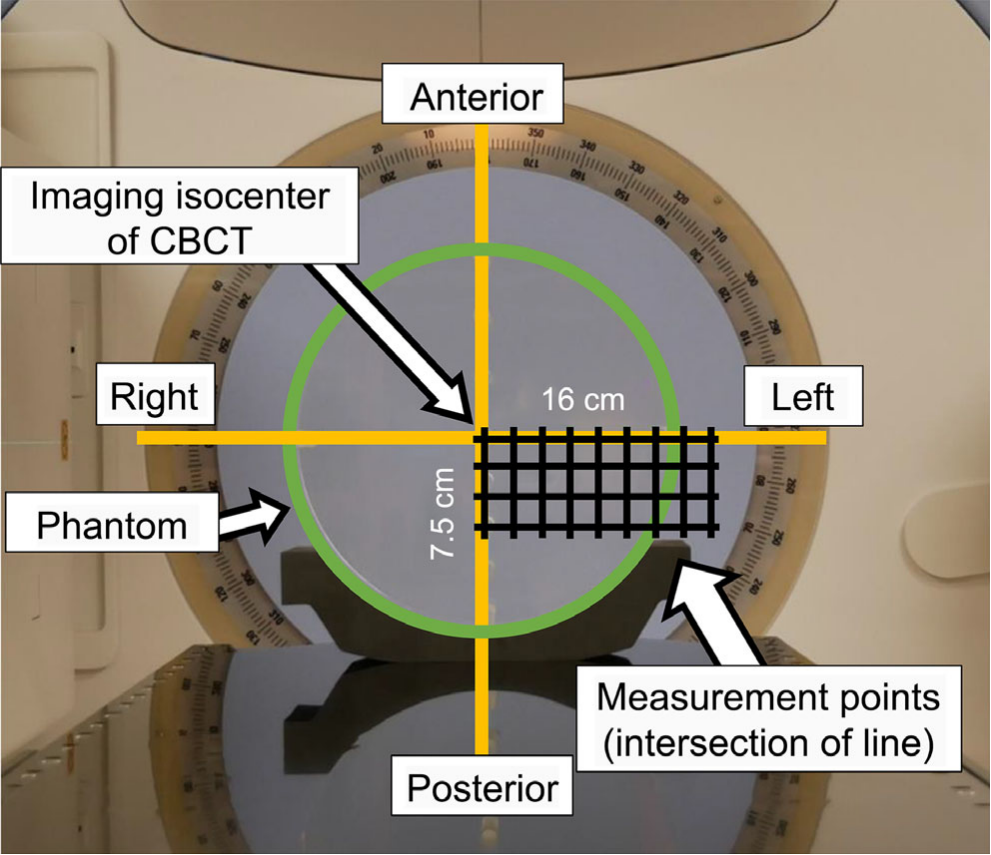
Measurement geometry of CTDI_w_. The *intersections* of the lines in the figure indicate the measurement points of CTDI_w_.

Figure 2 shows the measurement geometry of CTDI_w_. It was measured by placing the CTDI phantom center at 36 points at 2‐cm‐sized intervals from 0 (center) to 16 cm along the right–left (RL) direction and at 2.5‐cm‐sized intervals from 0 (center) to 7.5 cm along the anterior–posterior (AP) direction when assuming right breast irradiation. The intersections of the lines in Figure 2 indicate the CTDI_w_ measurement points. The maximum shift range for both horizontal and vertical directions from the isocenter of CBCT without the interaction of the gantry head and the couch during CBCT imaging was determined as the shift range of the phantom because of the patient's body size and the position of the dose reference point differ among patients.

### 2.E CTDI_w_ quantification

The measured air kerma was multiplied by the calibration factor and atmospheric correction factor to obtain CTDI 100 (CTDI_100_) values. CTDI_100_ is the value measured by inserting a pencil‐type ionization chamber dosimeter with a length of 100 mm into the measurement hole of the phantom for CTDI measurement. The calibration factor was obtained by comparing it with a dosimeter that was traceable to a Japanese national standard dosimeter. The measurement at each point was performed thrice, and the mean value of these measurements was used as the measured value. The extended uncertainty of the calibration factor was 6%, and the CTDI_100_ was calculated from the following equation:

(1) $${\text{CTDI}}_{100}=K_{a}\;\times \;k_{\mathrm{TP}}\;\times \;N,$$

where *K_a_* is the air kerma, *k*_TP_ is the atmospheric correction factor, and *N* is the calibration factor of the ionization chamber.

CTDI_w_ was calculated from the following equation:

(2) $${\text{CTDI}}_{w}=\frac{1}{3}{\text{CTDI}}_{100,c}+\frac{2}{3}{\text{CTDI}}_{100,p},$$

where CTDI_100,c_ is the CTDI_100_ at the center of the phantom and CTDI_100,p_ is the average of CTDI_100_ at the peripheral of the phantom. CTDI_w_ measurements were repeated by displacing the phantom along with the RL and AP directions from the isocenter of CBCT. The CTDI_100_ relative value against the value measured at the isocenter of CBCT at each measurement point was calculated using the following equation:

(3) $$\mathrm{Relative}\;\mathrm{value}\;\mathrm{of}\;{\mathrm{CTDI}}_{100}\;\left(\%\right)=({\mathrm{CTDI}}_{100}/{\mathrm{CTDI}}_{100,\;\mathrm{iso}})\;\times 100,$$

where CTDI_100,iso_ is the CTDI_100_ measured by placing the phantom at the isocenter of CBCT. The relative CTDI_w_ value was then calculated by displacing the phantom against the value measured at the isocenter of CBCT by using the following equation:

(4) $$\mathrm{Relative}\;\mathrm{value}\;\mathrm{of}\;{\mathrm{CTDI}}_{w}\;\left(\%\right)=({\mathrm{CTDI}}_{w}/{\mathrm{CTDI}}_{w,\;\mathrm{iso}})\;\times 100,$$

where CTDI_w,iso_ is the CTDI_w_ measured by placing the phantom at the isocenter of CBCT.

### 2.F Influence of human phantom position on right breast dose

**Fig. 3:**
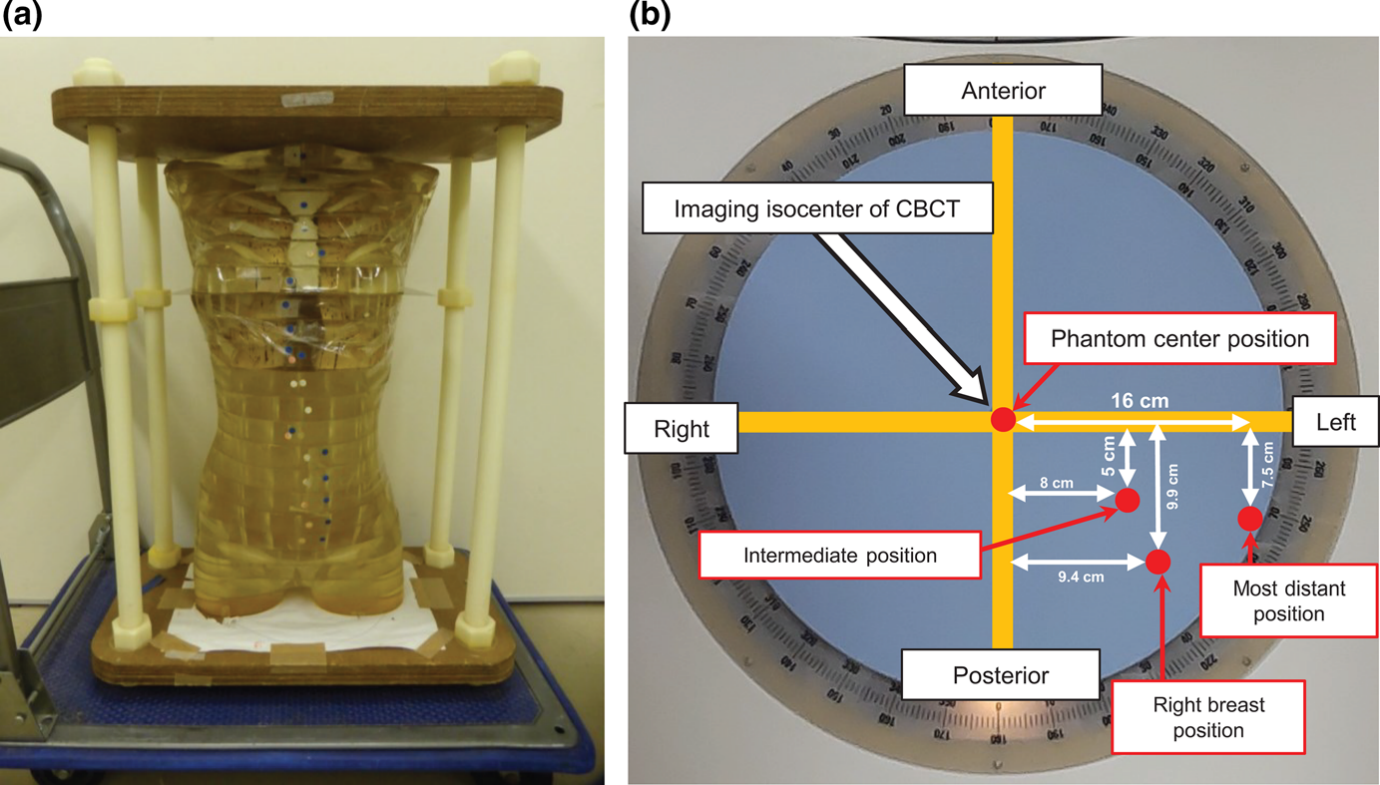
(a) Anthropomorphic acrylic phantom used in this study and (b) definitions of phantom positions.

The influence of human phantom position on right breast dose was investigated. The acquisition parameters for CBCT were the same as shown in Section 2.C. For this purpose, we used an acrylic human phantom containing human bones that simulates a human body. The absorbed doses in right breast were measured when the phantom was placed at the following four positions: the center of the phantom torso was placed at the isocenter of CBCT (phantom center position), the center of the phantom torso was placed 5 and 8 cm along the AP and RL directions, respectively, from the isocenter of CBCT (intermediate position), the center of the phantom torso was placed 7.5 and 16 cm along the AP and RL directions, respectively, from the isocenter of CBCT (most distant position), and the center of the phantom torso was placed 9.4 and 9.9 cm along the AP and RL directions, respectively, from isocenter of CBCT in order to adjust the right breast of the phantom to the isocenter of CBCT (right breast position). Figure 3 shows the anthropomorphic acrylic phantom used in this study and definitions of phantom positions.

Three optically stimulated luminescence (OSL) dosimeters (nanoDot; Landauer, Glenwood, IL, USA) were inserted into the center of the right breast in the phantom at each measurement. A 3‐mm acrylic plate was cut out to create a holder so that three OSL dosimeters were inserted adjacent to each other. The center of processed area for the insertion of the OSL dosimeters was aligned with the center of the right breast. After sandwiching the acrylic holder that the dosimeters were inserted, measurements were performed three times at each phantom position. Initialization of the OSL dosimeters was performed by irradiating them by visible light irradiated from a light emitting diodes for 3 hours, and initial values were read using a microStar reader (Landauer). The OSL dosimeters were calibrated with 80 kV x rays. Absorbed doses for the right breast (*D*_RB_) were calculated from the air kerma (*K*_a_) by the following equation:

(5) $$D_{\mathrm{RB}}=\left(K_{a}‐K_{\mathrm{ai}}\right)\frac{{\left(\mu_{\text{en}}/\rho \right)}_{\text{breast}}}{{\left(\mu_{\text{en}}/\rho \right)}_{\text{air}}},$$

where *K*_ai_ is the initial air kerma, (*μ*_en_/*ρ*)_breast_ is the mass energy absorption coefficient for breast (50% glandular tissue and 50% adipose tissue), and (*μ*_en_/*ρ*)_air_ is the mass energy absorption coefficient for air.^15^

## 3 RESULTS

**Fig. 4:**
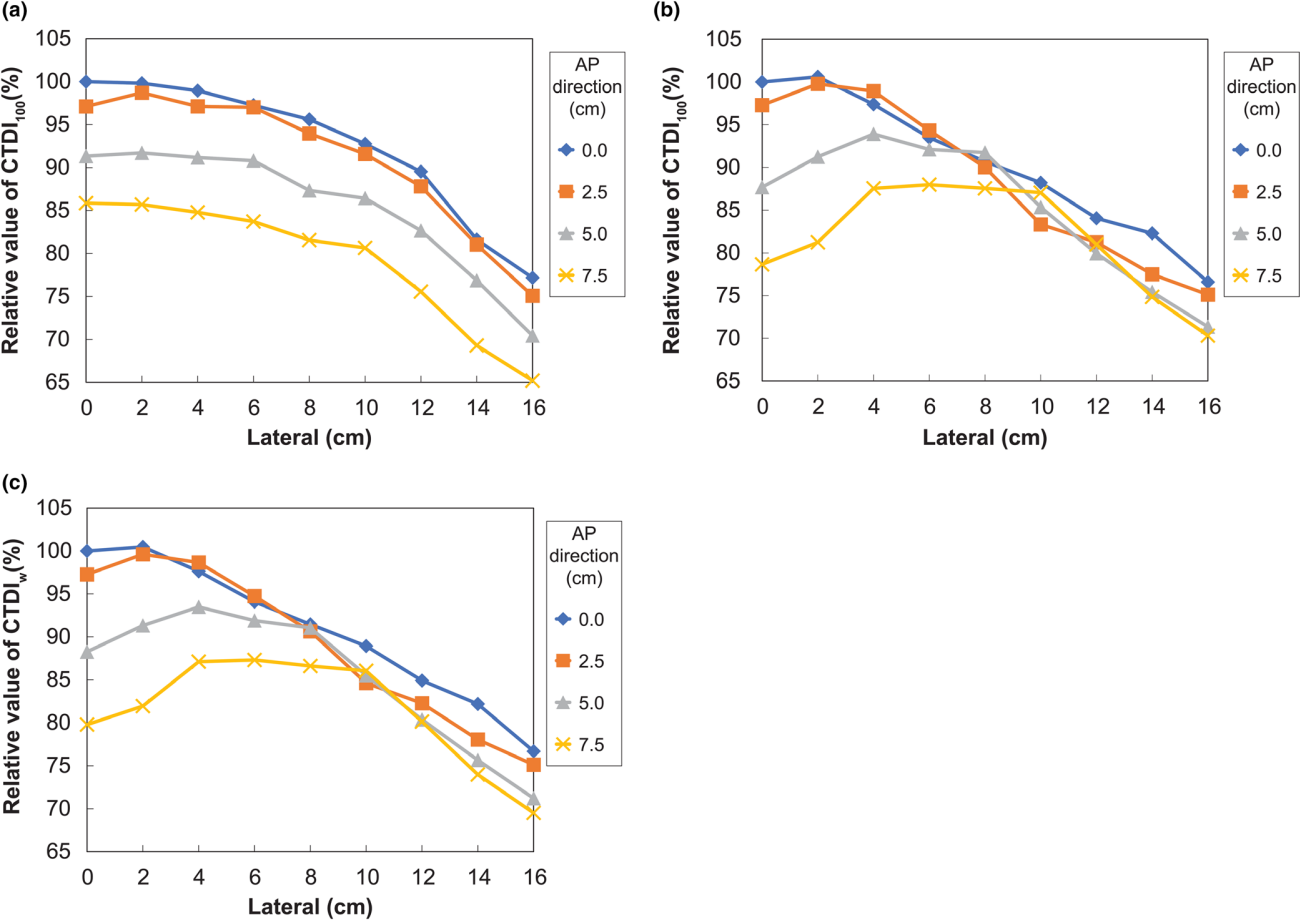
Relative values of CTDI_100_ and CTDI_w_ against the values measured at CBCT imaging center. The relative values of CTDI_100_ are shown for each position along the anterior–posterior (AP) direction: (a) relative values of CTDI_100_ at the center, (b) relative to the mean value of CTDI_100_ at peripheral sites, and (c) relative values of CTDI_w_.

Figure 4 shows the CTDI_100_ at the measurement hole in the center of the phantom, the average of the CTDI_100_ at the four measurement holes at the peripheral, and the CTDI_w_. The positive values in RL direction mean the phantom was moved from the isocenter to right direction, and those in AP direction mean the phantom was moved from the isocenter to posterior direction. The coefficient of variation of CTDI_w_ measured at the isocenter of CBCT was 0.57%. Nonuniform changes in CTDI_w_ were observed as the phantom moved from the isocenter of CBCT. The relative values of CTDI_w_ changed from 69.5% to 100.9% as the phantom deviated from the isocenter of CBCT along both the 90° and 180° directions. When the phantom deviated >5 cm or >10 cm along the AP and RL directions from the isocenter of CBCT, the relative value of CTDI_w_ reduced by >10% compared with that obtained at the isocenter of CBCT. The CTDI_w_ was ~30% lower than that obtained at the isocenter of CBCT when the phantom deviation was the largest, which was 7.5 and 16 cm at the AP and RL directions, respectively. The relative doses of CTDI_w_ were reported to be different according to the AP location at the measurement points up to 6 cm and converged at the measurement points >6 cm in the RL direction.

The absorbed doses for the right breast were 2.13 ± 0.15 mGy at the phantom center position, 2.06 ± 0.11 mGy at the intermediate position, 2.01 ± 0.12 mGy at the most distant position, and 1.96 ± 0.11 mGy at the right breast position. The relative values of the absorbed dose for the right breast, with that for the phantom center position being 100%, were 96.6% at the intermediate position, 94.4% at the most distant position, and 92.0% at the right breast position.

## 4 DISCUSSION

This study compared the CTDI_w_ of CBCT for IGRT with different phantom positions. CTDI_w_ was reduced by up to 30% when the center of the phantom was adjusted from the isocenter of CBCT to the irradiation center of the breast. Therefore, if the CTDI_w_ of CBCT for IGRT is measured at the isocenter of CBCT as well as diagnostic CT, there is a possibility to overestimate the CTDI_w_ measured at the patient location of radiation therapy. Thus, the deviated phantom locations contained a smaller amount of radiation compared with the isocenter of CBCT, and that the couch absorbed the x rays irradiated from the downward direction. Furthermore, CTDI_w_ seemed not to uniformly change because of the inhomogeneous dose distribution in the phantom. The change in dose distribution in the phantom is because the x‐ray beam used in this study had a narrow fan angle, and the range of the x‐ray irradiation angle was 215°. However, because of this angle, the x‐ray beam was not evenly irradiated to each measurement point in the phantom, which indicated that the amount of x‐ray attenuation by the couch changed according to its position. The relative values of CTDI_w_ with different locations can be used as correction factors to estimate the CTDI_w_ of the actual measurement location of the phantom. Therefore, the estimation accuracy of the effective dose can be improved using the effective dose conversion factor,^14^ and the DLP derived from CTDI_w_ that is measured at the patient location of radiation therapy. Although the effective dose does not represent the exposure of an individual patient, it is useful for comparing doses from different examinations.^16^ Consequently, Liao et al. measured CTDI_w_ in CBCT and calculated DLP from the CTDI_w_.^17^ Moreover, Abuhaimed et al. reported that DLP is useful for comparing patient doses between diagnostic CT and CBCT in radiation therapy.^18^ This study calculated DLP by multiplying the CTDI_w_ by the x‐ray beam width in a longitudinal direction. The effective dose can then be estimated using the DLP and the effective dose conversion factor. It may be overestimated up to ~30% if the CTDI_w_ is measured by placing the phantom at the isocenter of CBCT because the x‐ray beam width and effective dose conversion factor are constants and the effective dose only depends on the CTDI_w_. The effective dose calculated from DLP can be assumed to be the same trend as CTDI_w_. Certain studies have reported organ doses of CBCT in IGRT. However, little evidence shows organ doses of CBCT when the center of the patient’s torso was not adjusted to the isocenter.^5^, ^8^, ^9^, ^19^, ^20^ It is believed that the effective dose can be more precisely estimated by multiplying the relative value of CTDI_w_ by the CTDI_w_ measured at the isocenter of CBCT although CTDI is the radiation dose output of a CT scanner and cannot be used to compare patient dose.

Alvarado et al.^21^ reported organ doses when the center of the breast and the center of the trunk were located at the isocenter of CBCT, and the absolute dose of right breast were reduced by 10 to 23% when the center of the breast was located at the isocenter of CBCT compared with those when the center of the trunk was located at the isocenter of CBCT.

Their results showed similar trend with our results that the absorbed dose for the right breast decreased as the distance from the center of the phantom torso center and the isocenter of CBCT was enlarged, which were similar to CTDI results.

Note that this study has several limitations. The results of this study are for a single acquisition parameter for a specific device. Moreover, the results obtained in this study depend on the dose distribution by CBCT x‐ray tube. Therefore, the results are different when the rotation angle of the x‐ray tube is different. The x‐ray beam width along the longitudinal direction was wider than 40 mm. Therefore, the method for measuring CTDI in CBCT has room for improvement. Amer et al. proposed the cone beam CT dose index (CBDI)^13^ and Kim et al. compared several suitable methods for CBCT, including the CBDI.^12^ Moreover, the methods described in these studies were not used in the current study because this study only examined the influence of phantom positioning on the CTDI. The results of this study were based on CBCT apparatus used in this study and the irradiation conditions. However, additional studies are required because the results may be different when using other CBCT apparatuses. However, this study also showed that the effective dose may be overestimated if the CTDI_w_ measured at the isocenter is used when the center of the patient’s torso is not adjusted to the isocenter. Moreover, the outcome of this study is also postulated to help investigate the methods suitable for estimating patient doses in CBCT. In addition, the use of Monte Carlo simulations would enable dose estimation that reflects various imaging conditions and patients’ positions.

## 5 CONCLUSION

The measured CTDI_w_, which assumes breast irradiation, decreases by ~30% when the phantom deviates from the isocenter of CBCT compared with that measured at the isocenter of CBCT. The relative values of CTDI_w_ can be used as correction factors to estimate the CTDI_w_ of the actual measurement location that improves the estimation accuracy of effective dose when the patient is not set at the isocenter of CBCT.

This study showed that the dose of CBCT in IGRT should reflect the patient’s position. This may enable understanding the dose more accurately in actual situations.

## AUTHOR CONTRIBUTIONS

Hiroyuki Ueno was involved in conceptualization, data curation, investigation, visualization, and writing—original draft. Kosuke Matsubara and Akihiro Takemura were involved in supervision and writing—review and editing. Masato Hizume and Sayuri Bou were involved in writing—review and editing.

## CONFLICT OF INTEREST

The authors declare no conflict of interest.

## Data Availability

The data that support the findings of this study are available from the corresponding author upon reasonable request.
